# Apical membrane P2Y4 purinergic receptor controls K^+ ^secretion by strial marginal cell epithelium

**DOI:** 10.1186/1478-811X-3-13

**Published:** 2005-11-02

**Authors:** Daniel C Marcus, Jianzhong Liu, Jun Ho Lee, Elias Q Scherer, Margaret A Scofield, Philine Wangemann

**Affiliations:** 1 Cellular Biophysics Laboratory, Dept. Anatomy & Physiology, Kansas State University, Manhattan, KS 66506 USA; 2 Cell Physiology Laboratory, Dept. Anatomy & Physiology, Kansas State University, Manhattan, KS 66506 USA; 3 Molecular Pharmacology Laboratory, Dept. Pharmacology, Creighton School of Medicine, Omaha, NE 68178 USA

## Abstract

**Background:**

It was previously shown that K^+ ^secretion by strial marginal cell epithelium is under the control of G-protein coupled receptors of the P2Y family in the apical membrane. Receptor activation by uracil nucleotides (P2Y_2_, P2Y_4 _or P2Y_6_) leads to a decrease in the electrogenic K^+^ secretion. The present study was conducted to determine the subtype of the functional purinergic receptor in gerbil stria vascularis, to test if receptor activation leads to elevation of intracellular [Ca^2+^] and to test if the response to these receptors undergoes desensitization.

**Results:**

The transepithelial short circuit current (Isc) represents electrogenic K^+ ^secretion and was found to be decreased by uridine 5'-triphosphate (UTP), adenosine 5'-triphosphate (ATP) and diadenosine tetraphosphate (Ap4A) but not uridine 5'-diphosphate (UDP) at the apical membrane of marginal cells of the gerbil stria vascularis. The potencies of these agonists were consistent with rodent P2Y_4 _and P2Y_2 _but not P2Y_6 _receptors. Activation caused a biphasic increase in intracellular [Ca^2+^] that could be partially blocked by 2-aminoethoxy-diphenyl borate (2-APB), an inhibitor of the IP3 receptor and store-operated channels. Suramin (100 μM) did not inhibit the effect of UTP (1 μM). The ineffectiveness of suramin at the concentration used was consistent with P2Y_4 _but not P2Y_2_. Transcripts for both P2Y_2 _and P2Y_4 _were found in the stria vascularis. Sustained exposure to ATP or UTP for 15 min caused a depression of Isc that appeared to have two components but with apparently no chronic desensitization.

**Conclusion:**

The results support the conclusion that regulation of K^+ ^secretion across strial marginal cell epithelium occurs by P2Y_4 _receptors at the apical membrane. The apparent lack of desensitization of the response is consistent with two processes: a rapid-onset phosphorylation of KCNE1 channel subunit and a slower-onset of regulation by depletion of plasma membrane PIP_2_.

## Background

A high concentration of K^+ ^is maintained in the lumen of the cochlea via electrogenic secretion by the strial marginal cell epithelium [1]. One pathway of regulation is the coupling of purinergic receptors on the apical membrane of these cells to the apical potassium channels (IKs) which mediate secretion [2]. These receptors are responsive to both ATP and UTP as agonists and they were found to exert their action via the phospholipase C – protein kinase C intracellular signal pathway [3]. At the time of the original investigations, the purinergic receptor field recognized only 1 receptor responding to uracil nucleotides (P2U, now P2Y_2 _receptor). The known members of the mammalian metabotropic, G protein-coupled, purinergic receptors has since grown to P2Y_1_, P2Y_2_, P2Y_4_, P2Y_6_, P2Y_11_, P2Y_12_, P2Y_13 _and P2Y_14 _[4]. In addition to P2Y_2_, both the P2Y_4 _and P2Y_6 _receptors also respond to uracil nucleotides. On the basis of pharmacologic agonist and antagonist profiles, P2Y_6 _could be distinguished from P2Y_2 _and P2Y_4 _by the greater potency of UDP over UTP [5,6]. The pharmacologic distinction between P2Y_2 _and P2Y_4 _was more ambiguous and the accepted criteria changed rapidly [7].

The question of the subtype of P2Y receptor mediating regulation of K^+ ^secretion by strial marginal cells has been addressed by immunohistochemistry, which suggests the presence of P2Y_4 _in the apical marginal cell membrane [8]. However, a functional demonstration was lacking, the time-course of activation was not investigated and an independent demonstration of gene expression was not available.

The present study was conducted with the goals of 1) obtaining dose-response profiles for potentially definitive agonists of P2Y_2_, P2Y_4 _and P2Y_6 _receptors; 2) refining the profile by the use of antagonists, 3) testing for desensitization of the response to agonist and 4) determining the presence of transcripts for pyridine-sensitive P2Y receptors. The results support the conclusion that regulation of K^+ ^secretion by strial marginal cells occurs by apical P2Y_4 _receptors.

## Results

**Figure 1:**
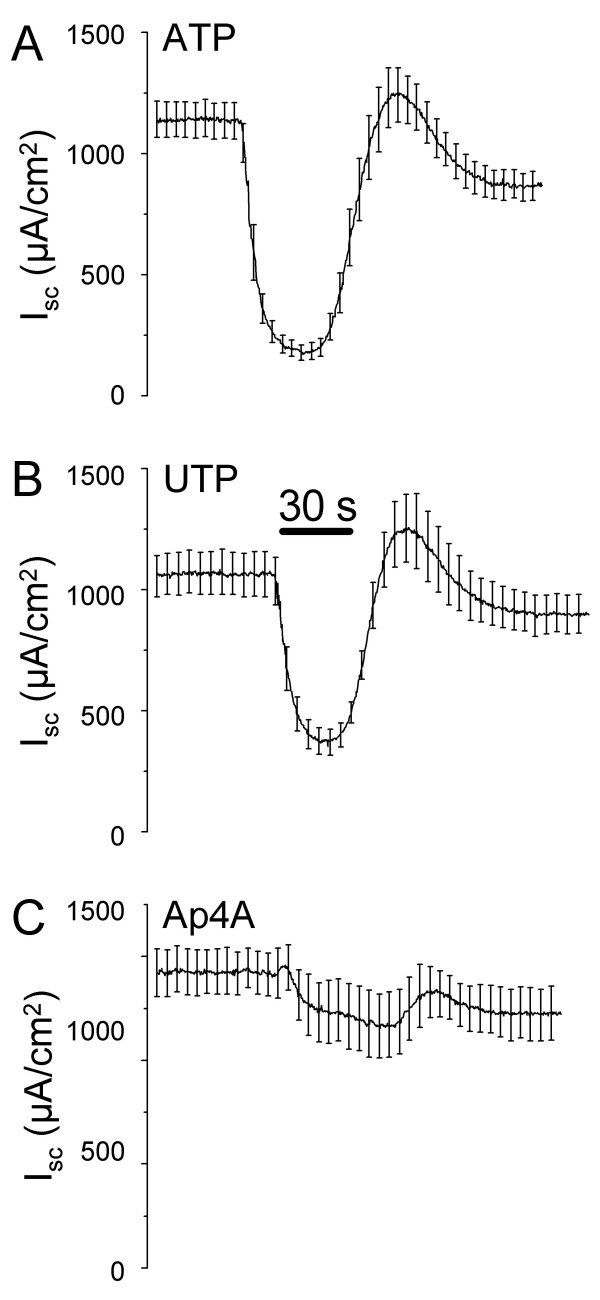
**Summary recordings of Isc (Ussing chamber) of strial marginal cells **during apical perfusion of A) ATP (10^-3 ^M; 30 s; n = 6), B) UTP (10^-4 ^M; 30 s; n = 7) and C) Ap4A (3 × 10^-4 ^M; 60 s; n = 4). Vertical bars are SEM (not all shown; spaced for clarity).

ATP perfused for 30 s at the apical side of strial marginal cell epithelium caused a monophasic decrease in *I*_*sc *_(Fig. 1A), consistent with previous findings [2]. Removal of ATP led to a recovery of *I*_*sc *_after an initial overshoot. Similar responses were seen from perfusion of other agonists, such as UTP and Ap4A (Fig. 1B &1C).

**Figure 2:**
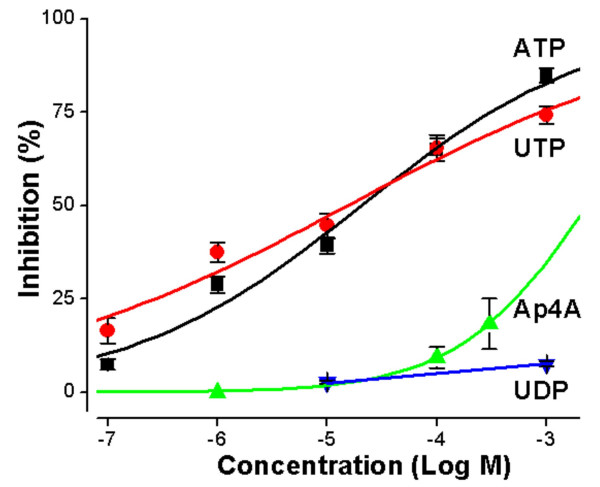
**Concentration-response curves for inhibition of Isc **(Ussing chamber) by P2 agonists. Mean ± SEM, n = 4 to 7 for each point. Data fit to Hill equation; fit parameters are given in the text.

The P2Y_6 _agonist, UDP, was tested after removal of contamination by UTP in the commercial product [6]. Contaminating UTP was digested by hexokinase in the presence of glucose. The enzymatically-purified UDP produced little effect on *I*_*sc *_(Fig. 2).

Concentration-response curves for the purinergic agonists are summarized in Figure 2. The potency order was UTP ≥ ATP > Ap4A >> UDP with EC_50 _values for UTP and ATP of about 2.3 and 1.2 × 10^-5 ^M. The relative potencies of the agonists are consistent with an action on P2Y_2 _and/or P2Y_4 _receptors.

**Figure 3:**
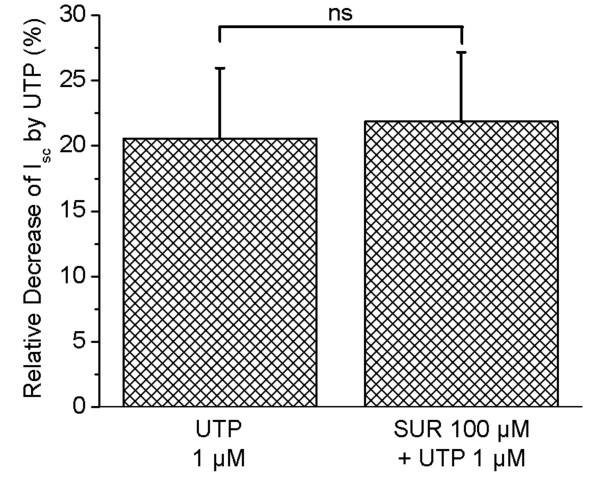
**Summary of relative decrease in Isc **(vibrating probe) caused by UTP (1 μM) in the absence or presence of suramin (100 μM).

The purinergic receptor antagonist, suramin, was tested for effectiveness in blocking the response to apical UTP. Suramin at 100 μM had no inhibitory effect on the action of the agonist, UTP (Fig. 3). UTP (1 μM) caused a decrease in *I*_*sc *_by 20.6 ± 5.4% in the absence of suramin and by 21.9 ± 5.3% (P > 0.05, n = 5) in the presence of suramin.

**Figure 4:**
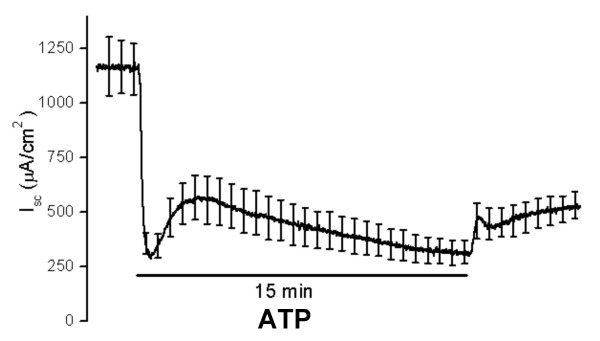
**Summary recordings of Isc **(Ussing chamber) of strial marginal cells during apical perfusion of ATP (10^-3 ^M) for 15 min. Vertical bars are SEM (not all shown; spaced for clarity; n = 6).

Both P2Y_2 _and P2Y_4 _receptor subtypes have been reported to undergo desensitization within 5–15 min [9,10]. Surprisingly, we found that sustained exposure (15 min) of the apical membrane to ATP led to a biphasic but sustained inhibition of *I*_*sc *_(Fig. 4). The recovery of *I*_*sc *_after removal of agonist was much slower, incomplete and without overshoot compared to acute exposure to agonist.

**Figure 5:**
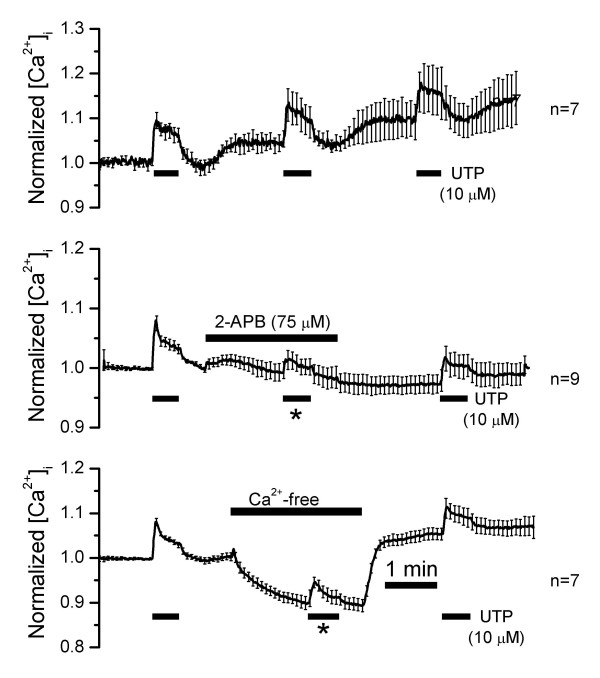
**Changes of intracellular Ca2+ concentration **([Ca^2+^]) of marginal cell layer in response to three consecutive perfusions of UTP (10 μM; 30 s). *Top*: Time control, no additional treatments (n = 7); *middle*: second exposure to UTP in the presence of the IP3 receptor inhibitor 2-APB (n = 9); *bottom*: second exposure to UTP in the absence of bath Ca^2+ ^(n = 7). Vertical bars are SEM (not all shown; spaced for clarity).

Perfusion of UTP (10 μM) for 30 s led to an increase in intracellular [Ca^2+^] that had both a peak and plateau phase and was repeatable (Fig. 5, *top*). Both the peak and plateau were significantly reduced by 2-APB (75 μM) (to 26.5 ± 3.9% and 25.1 ± 4.6%; Fig. 5, *middle*). 2-APB is an inhibitor of the IP3 receptor at this concentration [11,12] and can also inhibit other transporters including store-operated Ca^2+ ^channels [13]. Store operated channels, however, would not be activated under the present experimental conditions, since the Ca^2+ ^stores were not emptied prior to measurement. In the absence of external Ca^2+^, both the peak and plateau responses to UTP remained but were significantly reduced (to 68.9 ± 3.0% and 60.5 ± 4.2%; Fig. 5, *bottom*). The reductions, however, were likely the result of a general decrease in intracellular [Ca^2+^] seen after removal of bath Ca^2+ ^prior to perfusion of UTP. These findings taken together suggest that intracellular stores are the primary source of the increase in intracellular [Ca^2+^] induced by UTP.

**Figure 6:**
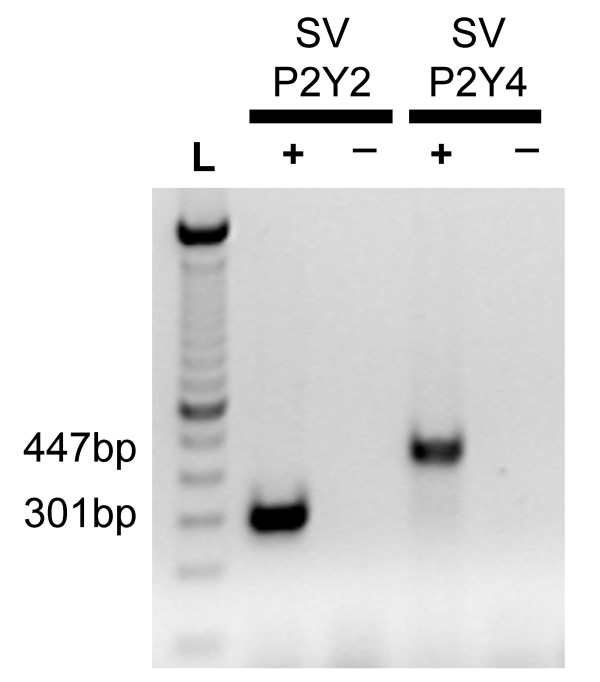
**Gel electrophoresis **of reverse transcription-polymerase chain reaction (RT-PCR) products from gerbil stria vascularis. Gene-specific primers were used for detection of transcripts for segments of P2Y_2 _and P2Y_4_. +, Reactions performed in the presence of reverse transcriptase; -, reactions performed in the absence of reverse transcriptase. Position of the bands for the expected lengths of the RT-PCR products [447 bp, P2Y_4_; 301 bp, P2Y_2_] are indicated and a 100-bp ladder is shown (L).

We tested for the presence of transcripts for P2Y_2 _and P2Y_4 _receptors in stria vascularis. Primers proven to recognize gerbil P2Y_2 _and P2Y_4 _[14] were used to amplify single, gene-specific bands in stria vascularis (Fig. 6); 301 base pairs (bp) for P2Y_2 _and 447 bp for P2Y_4_. Controls in which the reactions were run in the absence of reverse transcriptase (-RT) demonstrated the absence of contributions from genomic DNA. PCR products were analyzed for their sequence to confirm the identity of the bands. Sequences were the same as found previously in the gerbil vestibular labyrinth [14] (GenBank accession numbers: P2Y_2_, AF313448; P2Y_4_, AF313447).

## Discussion

Purinergic agonists control strial marginal cell K^+ ^secretion, a process observed as a transepithelial short circuit current, *I*_*sc *_[1,2]. Strial marginal cells secrete K^+ ^by a constellation of transporters previously described [1]. K^+ ^is taken up across the basolateral membrane by the Na^+^, K^+^-ATPase and the Na^+^, K^+^, 2Cl^-^-cotransporter. Na^+ ^carried into the cell on the cotransporter is removed by the Na^+^-pump and Cl^- ^carried into the cell on the cotransporter leaves by passive diffusion across a large Cl^- ^conductance in the basolateral membrane [15-17]. K^+ ^taken up by both the Na^+^-pump and cotransporter is secreted across the apical membrane by diffusion through IKs channels [18], consisting of KCNQ1 alpha subunits and KCNE1 beta regulatory subunits [19,20]. The epithelium regulates K^+ ^secretion by a variety of signal pathways, including those initiated by apical purinergic receptors, that converge at the IKs channel complex [21].

The previous demonstration of control of *I*_*sc *_in strial marginal cell epithelium by "P2U" receptors included determination of a concentration-response to ATP and comparative activities of other nucleotides (UTP, 2-MeS-ATP and α, β-meth-ATP) at 1 μM [2]. Full concentration-response curves were obtained in the present study to better define the active subtype. The lack of response to UDP eliminated P2Y_6 _as a candidate subtype [22-24]. The similar potency of ATP and UTP is consistent with hP2Y_2 _[23], rP2Y_2 _[22] but not hP2Y_4 _[6]. In fact, ATP acts as a competitive antagonist at the hP2Y_4 _receptor [25]. However, these results alone do not identify the strial marginal cell apical purinergic receptor as P2Y_2 _since it was subsequently found that rP2Y_4 _has an agonist potency sequence similar to rat and human P2Y_2 _[7,25]. Since the gerbil is expected to be more closely related to other rodents such as the rat, than to human, our finding is consistent with the contribution of P2Y_4 _and/or P2Y_2_.

The potency order of the agonists UTP, ATP, Ap4A and UDP in regulation of *I*_*sc *_from strial marginal cells is the same as for rodent P2Y_4 _receptors in other systems, including cloned mouse P2Y_4 _[26] and gerbil vestibular dark cells [2,27] even though the EC_50 _values are substantially shifted. The absolute values of EC_50_'s are alone not indicative of receptor binding for G protein-coupled receptors, although the relative potency remains constant. Receptor density plays a large role in defining the response of downstream effectors (IKs channels in this case) and differences in density can lead to significant shifts in EC_50 _curves.

Transcripts for both P2Y_2 _and P2Y_4 _were found in stria vascularis. The identity of the cell type(s) within the stria that contain these transcripts is not certain from these findings alone. Recent immunohistochemical findings show staining for the P2Y_4 _receptor at the apical membrane of strial marginal cells, while the antibody for P2Y_2 _stained at the basolateral region of the marginal cells and/or the intermediate cells [8], consistent with the functional evidence for apical P2Y_4 _shown here. The transcript expression confirms the immunohistochemical findings by an independent means and thereby provides an important verification of the specificity of the P2Y antibodies used in the previous study [8].

Ap4A was found to be a potent agonist at the hP2Y_2 _receptor [23] but much less potent than ATP at the hP2Y_4 _[28] and rP2Y_4 _receptors [24], similar to our finding in gerbil strial marginal cells. In spite of this similarity, uncertainty arises in the identification of the apical receptor in strial marginal cells as P2Y_4 _on this basis alone since there is also a conflicting report from Bogdanov et al. who found Ap4A to be equally potent as ATP at rP2Y_4 _receptors [7].

Suramin inhibits several P2 receptors, but it was found recently that at a high concentration (100 μM) it can be used to distinguish both rP2Y_2 _[22] and hP2Y_2 _[29] from both rP2Y_4 _[7] and hP2Y_4 _[29]. This criterion applied to the present results points to the apical P2Y purinergic receptor in gerbil strial marginal cells as the P2Y_4 _subtype. This conclusion based on function and pharmacology is consistent with the recent report of immunohistochemical localization of P2Y_4 _at the apical membrane of strial marginal cells [8]. We reported earlier that the response to ATP is increased in the absence of divalent cations [2]. This increased effect suggests that the apical receptor is preferentially activated by an uncomplexed form of ATP, as in aortic endothelial cells [30].

The overshoot of *I*_*sc *_after removal of purinergic agonists from the apical perfusate (Fig. 1) is most likely due to a release of K^+ ^accumulated in the epithelial cells during the inhibition of secretion across the apical membrane. The basolateral K^+ ^uptake mechanisms continue to operate for a time after inhibition of apical IKs channel complexes by activation of purinergic receptors, bringing the cytosolic K^+ ^concentration to a level higher in electrochemical potential above the apical bath than in the absence of agonist. Upon removal of agonist this "extra pool" of K^+ ^is suddenly released, resulting in the observed overshoot of *I*_*sc*_. The same phenomenon was reported previously [31] when K^+ ^secretion was first diminished by raising the apical K^+ ^concentration, thereby reducing the outward gradient across the apical cell membrane. Suddenly returning the apical perfusate K^+ ^concentration to the original low level led to an overshoot of *I*_*sc*_, as observed with the purinergic agonists.

The decrease in *I*_*sc *_observed in response to purinergic agonists could be due *a priori *to a reduction of electrogenic K^+ ^secretion but could also be accounted for by a stimulation of secretion of Cl^- ^or absorption of Na^+^. However, it was found in a previous study that the decrease in *I*_*sc *_could be completely accounted for by a decrease in K^+ ^secretion. Apical perfusion of 100 μM ATP lead to a decrease of *I*_*sc *_by 37.1 ± 3.7% and of K^+ ^secretory flux by 22.2 ± 5.5% [3].

The peak and plateau increases in intracellular [Ca^2+^] during perfusion of agonist in the presence and absence of bath Ca^2+^, as well as the reduction by 2-APB, are consistent with a release of Ca^2+ ^from intracellular stores. Ikeda et al. found a small monophasic increase in intracellular [Ca^2+^] of the entire stria vascularis that could not be analyzed further [32]. The same laboratory evaluated the purinergic response of cultured marginal cells and found agonist-induced increases in intracellular [Ca^2+^] that were not reduced upon removal of Ca^2+ ^from the bath [33]. Those cells were derived from guinea pig, the responses were monophasic and UTP was not tested as agonist.

The apparent absence of desensitization (Fig. 4) was a surprising finding in view of reports of rapid desensitization of both cloned P2Y_2 _and cloned P2Y_4 _purinergic receptors [9,10]. Desensitization has typically been observed as a decrease of inositol phosphate production and/or cytosolic Ca^2+ ^increase, either directly [34,35] or via the effect of Ca^2+ ^on transepithelial anion secretion [36]. Since the sustained response to UTP (Figure 4) is biphasic, it therefore may represent the summation of at least 2 processes.

The question arises as to the basis for the observed sustained decrease in IKs. Possible explanations include: 1) The P2Y_4 _receptor desensitizes several minutes after the onset of agonist, but the initial activation of P2Y_4 _leads to phosphorylation of KCNE1 via PKC [3] and the channel subunit remains phosphorylated due to inhibition of phosphatase activity. 2) Activation of P2Y_4 _leads to localized depletion of phosphatidylinositol-4,5-bisphosphate (PIP_2_) in the plasma membrane and to the consequent deactivation of the PIP_2_-dependent IKs channel.

*I*_*sc *_is controlled by apical P2Y_4 _receptors via the protein kinase C pathway by phosphorylation of the beta subunit of the IKs channel complex [3] [*vida infra*]. The sustained reduction in *I*_*sc *_and the incomplete recovery of *I*_*sc *_after prolonged exposure to agonist can both be explained if there is a decrease of phosphatase activity at the phosphorylation site. The channel subunit would then be expected to remain phosphorylated and the effect on *I*_*sc *_would be sustained even with a desensitization of the receptor. However, dephosphorylation rates have been reported to increase with elevated intracellular [Ca^2+^] in olfactory sensory neurons [37], arguing against this hypothesis.

The second explanation is more consistent with our findings. It has been found that activation of receptors (such as P2Y_4_) coupled to G_q/11 _deplete PIP_2 _[38] and that IKs (KCNQ1/KCNE1) activity is controlled by PIP_2 _[39]. In fact, the time course of desensitization of another K^+ ^channel by phenylephrine induced depletion of PIP_2 _via α_1_-adrenergic receptors is similar to the secondary effects seen here [38].

### Physiological significance

Purinergic receptors have been identified on the apical membrane of many of the cells forming the border of the cochlear duct [40-42]. Indeed, IKs of strial marginal cells in the cochlea is inhibited by UTP in much the same way as the homologous vestibular dark cells [2,17,27], although the lower potency of agonists on marginal cells suggests a lower density of receptors. The P2Y_4 _receptors can thereby provide the strial marginal cells with an autocrine as well as paracrine signaling pathway. Autocrine signaling is important for these cells since they have no gap junction communication [43], an unusual occurrence for epithelial cells. Paracrine signaling is also important in this organ since the rate of K^+ ^secretion must be adjusted for variations in K^+ ^efflux during stimulation of the cochlea by sound. In fact, the P2 receptors have been proposed to act as a mechanism to protect the inner ear from noise damage [44].

## Conclusion

The response to purinergic agonists at the apical side of strial marginal cells was shown here to be functionally mediated by P2Y4 receptors, previously shown to be present by immunostaining. The decrease in electrogenic K^+ ^secretion evoked by agonists was a) initially accompanied by a biphasic increase in intracellular Ca^2+ ^and b) surprisingly sustained in the continued presence of agonist. The sustained decrease in K^+ ^secretion was best explained by rapid-onset phosphorylation of KCNE1 channel subunit and a slower-onset of regulation by depletion of plasma membrane PIP_2_. Impaired PIP_2 _and IKs interaction was recently shown to be the basis of several mutations causing long QT syndrome, a genetic disease characterized by cardiac arrhythmias and deafness [45].

## Methods

### Animals and tissues

Gerbils (4–5 week old females) were anesthetized by injection of sodium pentobarbital (50 mg/kg, i.p.) and the temporal bones were removed. The method for dissecting strial marginal cell epithelium from the cochlear lateral wall was described previously [1]. Dissected epithelia were either transferred to a micro-Ussing chamber for measurement of the equivalent short circuit current (I_sc_), to a perfusion chamber for [Ca2+] measurement or were frozen in liquid nitrogen within 10 min of death for reverse transcription-polymerase chain reaction (RT-PCR). All procedures conformed to protocols approved by the Institutional Animal Care and Use Committee.

### Short-circuit current measurements

The micro-Ussing chamber for inner ear tissues has been described previously [3]. Briefly, the diameter of the aperture separating the apical and basolateral side half-chambers was 80 μm and each side was continuously perfused independently at 37°C with an exchange of solution accomplished within 1 s. *I*_*sc *_was measured with a 4-wire epithelial current clamp and recorded with a computer data acquisition system with 16 bits resolution. Samples were acquired at 32 Hz and decimated by a factor of 10. Perfusion changes were planned and carefully timed so that experiments from each experimental series could be averaged (Figures 1 &4). Results were analyzed and plotted using Origin software (OriginLab, Northampton, MA).

In one series of experiments (Figure 3), the relative *I*_*sc *_was measured with a vibrating probe as described previously [46]. Briefly, the lateral cochlear wall with stria vascularis was mounted in a perfusion chamber on the stage of an inverted microscope (Nikon TE-300) and continuously perfused with the same solution used for the micro-Ussing chamber at 37°C. The *I*_*sc *_relative to the initial control value was monitored by vibrating a platinum-iridium wire microelectrode coated with Pt-black on the tip. The probe was positioned 20–30 μm from the apical surface of the epithelium. The bath references were 26-gauge Pt-black electrodes. The probe tip was vibrated in the range of 200–400 Hz over an excursion of about 20 μm. Vibration between two positions within the line of current flow yields voltages in the low nanovolt range that correspond to current flow through the resistive physiological saline [47]. The output of the probe amplifier was recorded with ASET software (Science Wares, East Falmouth, MA). Although the single perfusate reached the entire stria vascularis, it was determined that brief changes of solution (≤30 s) effectively reached only the apical surface; the extremely tight junctions of the basal cell layer restricted diffusion of large molecules such as agonists and antagonists to the interior of the stria and to the basolateral membrane of marginal cells [1].

### Chemicals and solutions

In all experiments, both sides of the epithelium were perfused with a solution containing (in mM) NaCl 150, KH_2_PO_4 _0.4, K_2_HPO_4 _1.6, MgCl_2 _1, CaCl_2 _0.7, glucose 5, pH 7.4. All experimental agents were dissolved in this solution immediately before use. Suramin was purchased from Calbiochem (San Diego, CA) and adenosine 5'-triphosphate, 2-aminoethoxy diphenyl borate (2-APB), uridine 5'-triphosphate, uridine 5'-diphosphate (UDP), diadenosine tetraphosphate (Ap4A) and hexokinase from Sigma-Aldrich (St. Louis, MO). The 2-APB was predissolved in DMSO and used at a final DMSO concentration of 0.1%.

### Intracellular Ca^2+ ^measurements

Calcium was measured as described previously [48]. Briefly, stria vascularis was incubated for 20 min with the indicator dye 5 μM fluo-4-AM at 37°C (Molecular Probes, Eugene, OR) and mounted in the superfusion chamber on the stage of an inverted microscope (Diaphot, Nikon). The stria vascularis was folded into a loop with the marginal cell layer on the outside surface. An optical section of the marginal cells was observed and the recording slit, which defined the field of the Ca^2+ ^measurement, was restricted to this cell layer.

The preparation was alternately illuminated at 600 and 488 nm (Deltascan, Photon Technology International, South Brunswick, N.J.). The fluorescence signal was detected by a photon-counter (Photon Technology International) at a rate of 4 Hz. Changes in the emission intensity were taken as measures of changes in intracellular Ca^2+ ^([Ca^2+^]_i_). The drift of signal due to leakage of dye from the cells was linearly subtracted.

### Reverse transcription-polymerase chain reaction (RT-PCR)

Transcripts for P2Y_2 _and P2Y_4 _were assayed by RT-PCR using methods previously described for extraction of total RNA, DNase I treatment, PCR amplification, subcloning, and sequencing [14]. First strand cDNA synthesis was also performed as described previously [14] with the exception that 25 pmoles of random hexamers were used to prime the RNA. The sequence of the primers was based on the known sequences in the coding region of the rat, mouse and human receptors. P2Y_2 _primers: sense, 5'-GCTTCAACGAGGACTTCAAGTA(C/T)GTGC-3'; anti-sense, 5'-AGGTGAGGAAGAGGATGCTGCAGTAG-3'. P2Y_4 _primers: sense, 5'-CCAGAGGAGTTTGACCACTA-3'; anti-sense, 5'-CACCAAGGCCAGGGAGGA-3'. The primers were expected to yield RT-PCR products of 301 base pairs (bp) for P2Y_2 _and 447 bp for P2Y_4 _which were cloned and sequenced (GenBank accession numbers: P2Y_2_, AF313448; P2Y_4_, AF313447).

The PCR mixture was incubated as follows: 1 denaturation cycle for 5 min at 98°C; 40 amplification cycles consisting of denaturation for 45 sec at 95°C, annealing for 45 sec at 58°C, and extension for 45 sec at 72°C; and one extension cycle for 7 min at 72°C. PCR products were analyzed by horizontal electrophoresis in 2.0% agarose gels and visualized by ethidium bromide.

### Statistics

Data are expressed as the mean ± S.E.M. (*n *= number of tissues) of the I_sc _and concentration-response curves from changes in I_sc _were normalized to the response to 1 mM ATP. The Student's t-test of paired samples was used to determine statistical significance and increases or decreases in I_sc _were considered significant for *P *< 0.05.

## Competing interests

The author(s) declare that they have no competing interests.

## Authors' contributions

DCM conceived, designed and coordinated the study and drafted the manuscript. JL acquired the short-circuit current data with the Ussing chamber. JHL acquired the short-circuit current data with the vibrating probe. EQS acquired and analyzed the intracellular calcium data. MAS designed, performed and carried out the RT-PCR experiments. PW designed, interpreted and supported the intracellular calcium experiments.
